# Supplementary material for: The Microfluidic Toolbox for Analyzing Exosome Biomarkers of Aging
Source: Molecules. 2021 Jan 20;26(3):535. doi: 10.3390/molecules26030535 (PMC7864353; doi:10.3390/molecules26030535)
Supplement: Supplementary file 1 [file molecules-26-00535-s001.pdf]

molecules

Supplementary Materials

The Microfluidic Toolbox for Analyzing Exosome Biomarkers of Aging

Jonalyn DeCastro [1], Joshua Littig [1], Peichi Peggy Chou [2], Jada Mack-Onyeike [1], Amrita Srinivasan [3], Michael J. Conboy [4], Irina M. Conboy [4] and Kiana Aran [1,4,*]

[1] Keck Graduate Institute, The Claremont Colleges, Claremont, CA 91711, USA; jherce@kgi.edu (J.D.); jlittig18@students.kgi.edu (J.L.); jmack18@students.kgi.edu (J.M.-O.)
[2] Pitzer College, The Claremont Colleges, Claremont, CA 91711, USA; pechou@students.pitzer.edu
[3] Claremont McKenna College, The Claremont Colleges, Claremont, CA 91711, USA; asrinivasan23@students.claremontmckenna.edu
[4] Department of Bioengineering, University of California, Berkeley, CA 94720, USA; conboymj@berkeley.edu (M.J.C.); iconboy@berkeley.edu (I.M.C.)
* Correspondence: kiana_aran@kgi.edu

Table S1. Comparison of conventional techniques for exosome isolation, detection and characterization.

Isolation Technique	Basis of Isolation	Advantages	Limitations	Citations
**Ultracentrifugation or differential centrifugation**	Isolation based on size, shape, and particle density	Most used; standardized speeds; Vesicle concentration and enrichment; High sample purity; Subtype vesicle populations isolated with given speeds	Extensive assay time (<16 hrs); Low yield; Vesicle aggregation from high speeds; Potential contamination with soluble proteins and other macromolecules; Requires large and expensive equipment	[1-2]
**Filtration or ultrafiltration**	Isolation based on size and molecular weight (MWCO)	Yields higher concentration of exosomes; High sample purity; High intact exosome cargo (proteins and miRNA); Simple set up and equipment; Less time	Yields dilute samples; Potential loss of exosomes in membrane filter pores/clogging; Still requires a high-speed centrifuge	[3-4][3,4]
**Size-exclusion chromatography (SEC)**	Isolation based on size with gel-based chromatography	Yields highly pure samples; More uniform population size range; Ideal for small scale isolation; Pore sizes can match different exosome size ranges; Typically needs only low speed centrifugation step	Long extraction times; Difficult for large volumes; Dilute samples; Potentially low vesicle yield	[4-5]
**Polymer-based precipitation**	Isolation based on size with mesh-like polymeric precipitation	Exosomes pelleted at low centrifuge speeds; Uniform population size	Low purity of sample; Polymer precipitation excludes larger exosomes in sample; Single Exoquick kits are expensive; Limited to only a small number of samples	[4-5][4,5]
**Immunoaffinity**	Isolation based on immunoassays on beads or other surfaces	Higher target specificity; Secures exosome integrity; Techniques are simple and easy to use; Ability to isolate subpopulations with specific surface markers	Strong antibody-exosome interactions make elution difficult; Immuno-isolation devices require more expertise; Antibodies are costly; Other extracellular vesicles may have same surface markers	[1, 4]

Characterization Technique	Basis of Characterization	Advantages	Limitations	Citations
**Size-based characterization**				
**Dynamic light scattering**	Measure of scattered light upon incidence with particles of varying size	No sample preparation; Determines average size in monodisperse sample; Some software's can find zeta potential; Retain sample	Not suitable for polydisperse or heterogeneous samples; Only provides size	[6] [6]
**Nanoparticle tracking analysis**	Particles scatter laser beam upon incidence and motion is recorded by a CCD camera	Quick sample dilution prep; Determines size and concentration; Dynamic range within standard 10^{7}-10^{11}; Analysis of fluorescently labeled vesicles possible	Expertise for accurate optimization of camera and analysis settings required; No biochemical information of vesicles; Loss of sample in analysis	[6]
**Resistance pulse sensing**	Detection of single EV by transient decrease of ionic current	Tunable nanopores optimized for detection of different nanometer size ranges	Less widely used; Passing of electrical current in presence of vesicles; Lower LOD 100 nm	[6-8] [6–8]
**Flow cytometry**	EVs on beads illuminated by lasers in a flow chamber with hydrodynamic focusing	Small EVs (<500nm) detected collectively with swarm effect; High powered laser with specific modifications (CCD camera) for detection of smaller ~ 100 nm EVs<br>*Current development of single suspension and cytometry	Single EV detection only at above 500 nm; identify subpopulations of large EVs with specific fluorescent antigen fluorophores	[9-10]
**Morphology-based identification**				

**Transmission electron microscopy**	Uses electrons that pass-through sample for detection for 2D image of EVs	Images based on transparency of EV giving information about inner structures	Fixation, drying and vacuuming of sample (Very complicated); Electron beam can damage EVs	[10-11][10,11]
**Scanning electron microscopy**	Electron beam with detection of secondary electrons emitted by atoms in the area	Topography of EV surface; Size and morphology of EVs; Single EV morphology	Fixation, drying and vacuuming of sample (Very complicated); Electron beam can damage EVs	[10-11]
**Cryo-electron microscopy**	Imaging of ultra-thin vitrified film from flash freezing EVs in liquid nitrogen	High resolution imaging of EVs in native state without drying or vacuuming; Identification of specific subsets of EVs with immunogold labelling; Most accurate size determination; 3D tomography images possible	Can have high background noise; Equipment availability	[10-11][10,11]
**Atomic force microscopy**	EV interaction with tip of cantilever nanoprobe	Native EV sensing with minimal sample prep; Real 3D image of surface topography; Size and structural information of EVs; Gives info of mechanical EV properties like stiffness and elasticity	High resolution images require EVs be attached to atomically flat surface; EVs may change shape once bound to flat surface; Low through-put; Equipment availability	[10-11]
		**Biochemical EV Analysis**		
**Bradford assay or micro-bicinchoninic acid (BCA) assay**	Total protein concentration colorimetric assays	Easy to use; Simple standard colorimetric assay; Conventionally used as an estimation of EV concentration	General; Need highly pure EV sample; Protein contaminants can affect accuracy	[10][10]
**Immunosorbent Assays (ELISA)**	Use of specific EV antibodies to capture EVs on a supporting surface	Commonly done through ELISA with CD63, CD9 or CD81; Strong EV enrichment with capture; Capture antibodies can give subpopulations	Antibodies can be expensive and have different cross-reactivity; Typically for surface antigens unless do lysing step prior	[10]

**Immunoblotting**	Lysing of purified EVs and direct spotting on membrane (dot blot assay) or SDS-PAGE separation of proteins as in Western blot assay)	Commonly done through ELISA with CD63, CD9 or CD81 or cargo proteins ALIX, TSG101	Only semi quantitative and limited to bulk assays; Does not provide on heterogeneity of sample; Requires large volumes and extensive sample processing	[10]

Table S2. Comparative analysis and specifications of microfluidic devices for exosome isolation and detection.

Device name	Sample Type	Input Volume	Method	Data Output	Limit of Detection	Sensitivity	Yield	Time	Citation
**Exosome Isolation**									
Field-Based Isolation									
Acoustofluidics	Human blood	100 µL	Acoustics	Separation of primary human trophoblast-derived EVs and MVs; isolation of >150 nm particles	N/A	N/A	~82%	25 min	[12]
Asymmetric flow field-flow fractionation (AF4)	Melanoma-derived EVs	N/A	Separation based on particle density and hydrodynamic properties	Separation of large (90-120 nm) and small (60-80 nm) exosomes and ~35 nm nanoparticles. real-time dynamic light scattering (DLS) measurements	N/A	N/A	N/A	A few hours	[13]
Thakur et al. microfluidic device	A-549 cells, SH-SY5Y cells, blood serum, urine from a lung cancer model	N/A	Localized surface plasmon resonance	Detection and distinguishment of exosomes from multivesicular vesicles	0.194 µg/mL	0.01793 µg/mL	N/A	30 min	[14]
RInSE	Cell culture supernatant	~ 20 uL	Rapid inertial solution exchange + immunolabeling on magnetic beads	Characterization of EPCAM cancer marker; Exosome diameter; RNA fluorescence measurements	N/A	N/A	99%	4-5 hrs	[15]
Eletrophoretic system	Plasma	1000 µL/h (500 µL for tests)	electrical migration and size exclusion	Zeta potential shift; diameter size	N/A	N/A	65%	30 min	[16]
Surface functionalized Isolation									
Xia et al. microfluidic device	Serum from breast cancer patients	N/A	Carbon nanotubes with anti-CD63; Colorimetric assay	Colorimetric detection	5.2×10^5 particles/ µL	N/A	N/A	40 min	[17]
Zn-O Chip	Blood serum	100 µL	Surface functionalization w/ anti-CD63 Ab; TMB-based colorimetric assay	Colorimetric detection	2.2×10^4 particles/ µL	2.2×10^5 - 2.4×10^7 particles/ µL	N/A	N/A	[18]
BAF-TiN Biosensor	Serum-derived exosomes	N/A	Immunocapture	Raman investigation of biotin direct adsorption on TiN film. And atomic	4.29×10^{-3} µg mL^{-1}	0.005-500 µg/mL	N/A	2200s (36.7min)	[19]

	from mice			force microscopy to detect anti-CD63 antibody.	for CD63, an exosome marker, and 2.75 × 10−3 μg mL−1 for epidermal growth factor receptor variant-III,				
gFET biosensor	Lyophilized exosomes	10 uL	gFET surface functionalization w/ anti-CD63 Ab	Real-time current and voltage measurements; exosome concentration	0.1 ug/ml	5000 exosomes/ μL	N/A	30 min	[20]
Doldan et al. microfluidic device	N/A	1.5 uL	Surface functionalization + electrochemical sensing	Real-time current measurements; exosome concentration	2x10^2 particles/uL	N/A	N/A	N/A	[21]
An integrated double-filtration microfluidic device	Urine of bladder cancer patients	10 μL of plasma per marker	Size-exclusion filtration	ELISA	N/A	81.3%	74.2%	N/A	[22]
Integrated Magneto−Electrochemical exosome (iMEX) platform	Plasma or serum	10 μL	magnetic enrichment and enzymatic amplification,	Current change over time	3 × 104 exosomes per sample (10 μL)	~10^5 vesicles with 10 μL of samples	N/A	1 hour	[23]
Surface Plasmon Resonance Platform	Serum and exosomes from BT474 breast cancer cell line.	N/A	Surface plasmon resonance	Spectral shift analysis of captured exosomes	2070 exosomes/ μL	2.07x10^3 to 3.3x10^4 exosomes/uL	N/A	N/A	[24]
Alternating Current Electrokinetic (ACE) Microarray Chip	Undiluted plasma spiked with glioblastoma exosomes. Also whole blood	30−50 μL	Dielectrophoretic separation force; immunofluorescence	Flourescence analysis of exosomes, EVs, and RNA and immunoflourescence analysis of CD 63 and TSG101 proteins	N/A	N/A	N/A	>30 min	[25]

ExoTENPO	Murine and clinical cohort exosomes in serum and plasma	N/A	Magnetically trapping and sorting magnetically labeled exosomes	Dynamic light scattering; exosome concentration; RNA; machine learning	N/A	N/A	N/A	10mL/h	[26]
Liu et al. microfluidic device	Plasma; Cell culture supernatant	50 uL	Surface plasmon resonance w/ gold film	Retractive index; Exosome concentration with specific antibody binding	2 × 10^10 exosomes/mL	9.258 x 10^3 %/RIU	N/A	N/A	[27]
Plasmonic interferometer array	Lung cell culture supernatant	N/A	Plasmonic interferometer array (PIA)	Refractive index unit	3.86×10^8 exosomes/mL	9.72×10^9 exosomes/mL (smartphone detection)	N/A	N/A	[28]
Oh et al. Microfluidic assay	Bovine serum cultured with neuroblastoma and cervical cancer cells.	N/A	In vitro microfluidic cell culture assay	Immunofluorescent stains, NTA with Nanosight, RT-PCR	N/A	N/A	N/A	N/A	[29]
SAW-IEM Chip	Untreated plasma	20 uL	Surface acoustic waves and ion exchange membranes	Mechanism exosome lysing; miRNA quantification	1pM	N/A	N/A	20 min	[30]

References

1. Contreras-Naranjo, J.C.; Wu, H.-J.; Ugaz, V.M. Microfluidics for exosome isolation and analysis: enabling liquid biopsy for personalized medicine. *Lab a Chip* **2017**, *17*, 3558–3577, doi:10.1039/c7lc00592j.
2. Chiriacò, M.S.; Bianco, M.; Nigro, A.; Primiceri, E.; Ferrara, F.; Romano, A.; Quattrini, A.; Furlan, R.; Arima, V.; Maruccio, G. Lab-on-Chip for Exosomes and Microvesicles Detection and Characterization. *Sensors* **2018**, *18*, 3175, doi:10.3390/s18103175.
3. He, M.; Zeng, Y. Microfluidic Exosome Analysis toward Liquid Biopsy for Cancer. *J. Lab. Autom.* **2016**, *21*, 599–608, doi:10.1177/2211068216651035.
4. Yu, L.-L.; Zhu, J.; Liu, J.-X.; Jiang, F.; Ni, W.-K.; Qu, L.-S.; Ni, R.-Z.; Lu, C.-H.; Xiao, M. A Comparison of Traditional and Novel Methods for the Separation of Exosomes from Human Samples. *BioMed Res. Int.* **2018**, *2018*, 1–9, doi:10.1155/2018/3634563.
5. Stranska, R.; Gysbrechts, L.; Wouters, J.; Vermeersch, P.; Bloch, K.; Dierickx, D.; Andrei, G.; Snoeck, R. Comparison of membrane affinity-based method with size-exclusion chromatography for isolation of exosome-like vesicles from human plasma. *J. Transl. Med.* **2018**, *16*, 1–9, doi:10.1186/s12967-017-1374-6.
6. Witwer, K.W.; Buzás, E.I.; Bemis, L.T.; Bora, A.; Lässer, C.; Lötvall, J.; Hoen, E.N.N.- 'T; Piper, M.G.; Sivaraman, S.; Skog, J.; et al. Standardization of sample collection, isolation and analysis methods in extracellular vesicle research. *J. Extracell. Vesicles* **2013**, *2*, doi:10.3402/jev.v2i0.20360.
7. de Vrij, J. et al. Quantification of nanosized extracellular membrane vesicles with scanning ion occlusion sensing. Nanomedicine (Lond) 8, 1443–1458 (2013).
8. Momen-Heravi, F.; Balaj, L.; Alian, S.; Etigges, J.; Etoxavidis, V.; Ericsson, M.; Distel, R.J.; Ivanov, A.R.; Skog, J.; Kuo, W.P. Alternative Methods for Characterization of Extracellular Vesicles. *Front. Physiol.* **2012**, *3*, 354, doi:10.3389/fphys.2012.00354.
9. Gurunathan, S., Kang, M.-H., Jeyaraj, M., Qasim, M. & Kim, J.-H. Review of the Isolation, Characterization, Biological Function, and Multifarious Therapeutic Approaches of Exosomes. Cells 8, (2019).
10. Hartjes, T.A.; Mytnyk, S.; Jenster, G.; Van Steijn, V.; Van Royen, M.E. Extracellular Vesicle Quantification and Characterization: Common Methods and Emerging Approaches. *Bioeng.* **2019**, *6*, 7, doi:10.3390/bioengineering6010007.
11. Szatanek, R.; Baj-Krzyworzeka, M.; Zimoch, J.; Lekka, M.; Siedlar, M.; Baran, J. The Methods of Choice for Extracellular Vesicles (EVs) Characterization. *Int. J. Mol. Sci.* **2017**, *18*, 1153, doi:10.3390/ijms18061153.
12. Wu, M.; Ouyang, Y.; Wang, Z.; Zhang, R.; Huang, P.-H.; Chen, C.; Li, H.; Li, P.; Quinn, D.; Dao, M.; et al. Isolation of exosomes from whole blood by integrating acoustics and microfluidics. *Proc. Natl. Acad. Sci.* **2017**, *114*, 10584–10589, doi:10.1073/pnas.1709210114.
13. Zhang, H.; Freitas, D.; Kim, H.S.; Fabijanic, K.; Li, Z.; Chen, H.; Mark, M.T.; Molina, H.; Benito-Martin, A.; Bojmar, L.; et al. Identification of distinct nanoparticles and subsets of extracellular vesicles by asymmetric flow field-flow fractionation. *Nat. Cell Biol.* **2018**, *20*, 332–343, doi:10.1038/s41556-018-0040-4.
14. Thakur, A. et al. Direct detection of two different tumor-derived extracellular vesicles by SAM-AuNIs LSPR biosensor. Biosens Bioelectron 94, 400–407 (2017).
15. Dudani, J.S.; Gossett, D.R.; Tse, H.T.K.; Lamm, R.J.; Kulkarni, R.P.; Di Carlo, D. Rapid inertial solution exchange for enrichment and flow cytometric detection of microvesicles. *Biomicrofluidics* **2015**, *9*, 014112, doi:10.1063/1.4907807.
16. Cho, S.; Jo, W.; Heo, Y.; Kang, J.Y.; Kwak, R.; Park, J. Isolation of
17. Xia, Y.; Liu, M.; Wang, L.; Yan, A.; He, W.; Chen, M.; Lan, J.; Xu, J.; Guan, L.; Chen, J. A visible and colorimetric aptasensor based on DNA-capped single-walled carbon nanotubes for detection of exosomes. *Biosens. Bioelectron.* **2017**, *92*, 8–15, doi:10.1016/j.bios.2017.01.063.
18. Chen, Z.; Cheng, S.-B.; Cao, P.; Qiu, Q.-F.; Chen, Y.; Xie, M.; Xu, Y.; Huang, W.-H. Detection of exosomes by ZnO nanowires coated three-dimensional scaffold chip device. *Biosens. Bioelectron.* **2018**, *122*, 211–216, doi:10.1016/j.bios.2018.09.033.
19. Qiu, G. et al. Detection of Glioma-Derived Exosomes with the Biotinylated Antibody-Functionalized Titanium Nitride Plasmonic Biosensor. Advanced Functional Materials 29, 1806761 (2019).
20. Chemically Functionalised Graphene FET Biosensor for the Label-free Sensing of Exosomes | Scientific Reports. https://www.nature.com/articles/s41598-019-50412-9.

21. Doldán, X.; Fagúndez, P.; Cayota, A.; Laíz, J.; Tosar, J.P. Electrochemical Sandwich Immunosensor for Determination of Exosomes Based on Surface Marker-Mediated Signal Amplification. *Anal. Chem.* **2016**, *88*, 10466–10473, doi:10.1021/acs.analchem.6b02421.
22. Liang, L.-G.; Kong, M.-Q.; Zhou, S.; Sheng, Y.-F.; Wang, P.; Yu, T.; Inci, F.; Kuo, W.P.; Li, L.-J.; Demirci, U.; et al. An integrated double-filtration microfluidic device for isolation, enrichment and quantification of urinary extracellular vesicles for detection of bladder cancer. *Sci. Rep.* **2017**, *7*, srep46224, doi:10.1038/srep46224.
23. Jeong, S.; Park, J.; Pathania, D.; Castro, C.M.; Weissleder, R.; Lee, H. Integrated Magneto–Electrochemical Sensor for Exosome Analysis. *ACS Nano* **2016**, *10*, 1802–1809, doi:10.1021/acsnano.5b07584.
24. Sina, A. A. I. et al. Real time and label free profiling of clinically relevant exosomes. Scientific Reports 6, 30460 (2016
25. Ibsen, S.D.; Wright, J.; Lewis, J.M.; Kim, S.; Ko, S.-Y.; Ong, J.; Manouchehri, S.; Vyas, A.; Akers, J.; Chen, C.C.; et al. Rapid Isolation and Detection of Exosomes and Associated Biomarkers from Plasma. *ACS Nano* **2017**, *11*, 6641–6651, doi:10.1021/acsnano.7b00549.
26. Ko, J.; Bhagwat, N.; Yee, S.S.; Ortiz, N.; Sahmoud, A.; Black, T.; Aiello, N.M.; McKenzie, L.; O'Hara, M.; Redlinger, C.; et al. Combining Machine Learning and Nanofluidic Technology To Diagnose Pancreatic Cancer Using Exosomes. *ACS Nano* **2017**, *11*, 11182–11193, doi:10.1021/acsnano.7b05503.
27. Liu, C.; Zeng, X.; An, Z.; Yang, Y.; Eisenbaum, M.; Gu, X.; Jornet, J.M.; Dy, G.K.; Reid, M.E.; Gan, Q.; et al. Sensitive Detection of Exosomal Proteins via a Compact Surface Plasmon Resonance Biosensor for Cancer Diagnosis. *ACS Sensors* **2018**, *3*, 1471–1479, doi:10.1021/acssensors.8b00230.
28. Zeng, X.; Yang, Y.; Zhang, N.; Ji, D.; Gu, X.; Jornet, J.M.; Wu, Y.; Gan, Q. Plasmonic Interferometer Array Biochip as a New Mobile Medical Device for Cancer Detection. *IEEE J. Sel. Top. Quantum Electron.* **2019**, *25*, 1–7, doi:10.1109/jstqe.2018.2865418.
29. Oh, H.J.; Shin, Y.; Chung, S.; Hwang, D.W.; Lee, D.S. Convective exosome-tracing microfluidics for analysis of cell-non-autonomous neurogenesis. *Biomater.* **2017**, *112*, 82–94, doi:10.1016/j.biomaterials.2016.10.006.
30. Ramshani, Z.; Zhang, C.; Richards, K.; Chen, L.; Xu, G.; Stiles, B.L.; Hill, R.; Senapati, S.; Go, D.B.; Chang, H.-C. Extracellular vesicle microRNA quantification from plasma using an integrated microfluidic device. *Commun. Biol.* **2019**, *2*, 1–9, doi:10.1038/s42003-019-0435-1.
